# Outcomes of Patients With Familial Central Precocious Puberty due to Mutations of *MKRN3* Gene After Treatment With Gonadotropin-Releasing Hormone Agonist

**DOI:** 10.1155/ije/5609749

**Published:** 2025-11-21

**Authors:** Ziwei Chen, Wenying Li, Junqi Wang, Zhiya Dong, Chuanyin Li, Wei Wang, Ronggui Hu, Xiaoyu Ma, Yuan Xiao, Wenli Lu

**Affiliations:** ^1^Department of Pediatrics, Ruijin Hospital Affiliated to Shanghai Jiao Tong University, Shanghai, China; ^2^Cancer Center, Shanghai Tenth People's Hospital, School of Medicine, Tongji University, Shanghai, China

**Keywords:** familial central precocious puberty, GnRHa therapy, gonadal development, *MKRN3* mutations

## Abstract

**Objective:**

To assess the therapeutic effects of gonadotropin-releasing hormone agonist (GnRHa) on children with familial central precocious puberty (FCPP) due to Makorin ring finger Protein 3 (*MKRN3*) gene mutations.

**Methods:**

Children with central precocious puberty (CPP) who were admitted to the Pediatric Endocrinology Department of Shanghai Ruijin Hospital from 2014 to 2021 were enrolled, of whom 4 FCPP children with MKRN3 gene mutations, including 3 girls and 1 boy, were selected as research subjects. Their height, weight, body mass index (BMI), predicted adult height (PAH), bone age, bone age advance (BAA, bone age minus chronological age), height-based standard deviation scores (Ht-SDS) corresponding to the chronological age, concentrations of sex hormones (luteinizing hormone [LH] and follicle-stimulating hormone [FSH]), and development of sexual organs were compared before and after at least 2 years of GnRHa treatment.

**Results:**

After at least 2-year GnRHa treatment, mean volume of uterus of three girls decreased from 5.72 ± 2.58 to 2.12 ± 1.62 mL (*p* < 0.05) and mean volume of ovaries decreased from 3.61 ± 1.67 to 0.62 ± 0.22 mL (*p* < 0.05) as well, indicating that the gonadal development was effectively inhibited. Basal concentrations of LH and FSH in serum decreased, indicating that the secretion of gonadotropin in the anterior pituitary is inhibited. BAA and Ht-SDS decreased, suggesting that the bone age was restrained, and the growth rate was slowed down to some extent. Both average BMI and obesity prevalence (*X*^2^ = 7.188, *p*=0.029) decreased during the treatment. No obvious adverse reaction was found.

**Conclusion:**

Long-term GnRHa treatment could effectively inhibit the gonadal development and FSH secretion in FCPP children with *MKRN3* gene mutations, while this inhibitory effect on the bone age and growth rate was not obvious. Adverse reactions such as increased prevalence of obesity were not found. A large-scale, long-term follow-up study is required to indicate whether patients' final height (FH) could reach PAH or target height (TH).

## 1. Introduction

Puberty is an essential biological process defined as “the period of the first becoming capable of reproducing,” which is marked by maturation of genital organs, development of secondary sexual characteristics, acceleration of growth velocity (GV), changes in effect, and the occurrence of menarche in women [1]. Puberty is regulated by genetic and environmental factors and initiated by an increase in pulsatile secretion of gonadotropin-releasing hormone (GnRH) that is released from hypothalamic neuroendocrine neurons [2]. Early activation of the hypothalamic-pituitary-gonadal (HPG) axis results in central precocious puberty (CPP), which is marked by thelarche prior to 8 years in girls or testicular enlargement prior to 9 years in boys [3]. CPP can be secondary to the central nervous system (CNS) tumors or lesions, while it is dominantly idiopathic. A previous research has shown that a quarter of idiopathic central precocious puberty (ICPP) can be familial central precocious puberty (FCPP) [4]. A portion of genetic causes of FCPP has been found over the past decades, such as mutations of Makorin ring finger Protein 3 (*MKRN3*) and delta-like 1 homolog (DLK1). MKRN3 is an intronless gene located inside a region containing an imprinted gene cluster at chromosome 15q11-q13. It undergoes maternal imprinting, therefore, only the paternal allele is expressed, while the maternal allele is silenced through methylation of CpG islands. MKRN3 function is associated with gene transcription and E3 ubiquitin ligase activity [5]. Previous studies on mice have shown that MKRN3 mRNA is highly expressed in the hypothalamus arcuate nucleus during infancy and early adolescence, decreases at the beginning of adolescence, and then, remains at a low level [6], which suggested that MKRN3 may be associated with the inhibition of GnRH secretion before puberty [7]. Inactivating *MKRN3* mutations were first described by Abreu et al. in 2013 in 15 patients from 5 different families with CPP [6]. Thereafter, diverse types of *MKRN3* mutations have been reported, and MKRN3 defects have been recognized as the most common monogenic cause of FCPP.

Long-acting GnRHa has been the first-line treatment for all etiologies, as a long-term study on the treatment of CPP with GnRHa was originally published in 1986 by Comite. It was found that GnRHa could effectively slow down the growth rate, control the progression of the bone age, and improve predicted adult height (PAH) [8]. There is no evidence that long-term GnRHa may lead to adverse reactions, such as functional disturbance of gonad, excessive obesity, and decreased bone mineral density [9]. After that, GnRHa has been the main treatment for CPP. In addition, novel treatment agents (e.g., histrelin subdermal implant and leuprolide acetate) have been widely utilized [10].

**Figure 1:**
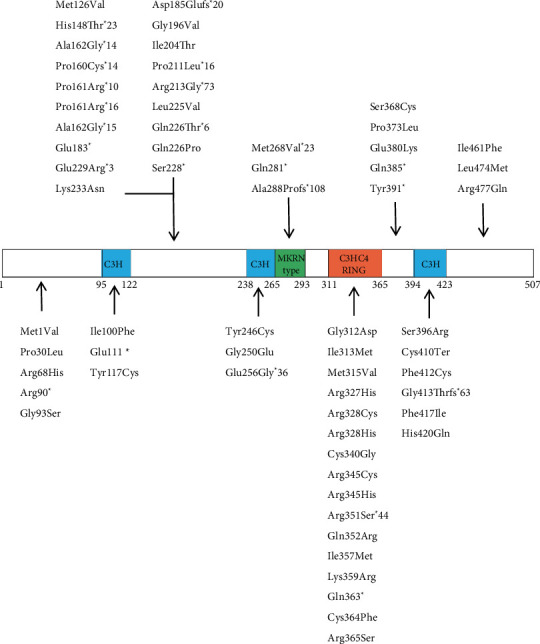
The reported MKRN3 mutations in human with CPP.

By March 2024, a total of 69 types of *MKRN3* mutations were found in 152 CPP patients [5, 10–21], including 63 coding region mutations and 6 5′-UTR mutations. Among the coding region mutations, 39 were missense mutations (accounting for 62%), 18 were frameshift mutations, and 6 were nonsense mutations. These sites are not scattered in MKRN3 but mainly concentrated in several specific functional domains (including zinc finger structure domain, ring finger domain, and Makorin region), as shown in Figure 1.

A multiethnic cohort study described the clinical features and hormone concentrations of children with CPP caused by mutations in *MKRN3* and compared different types of mutations [5]. This study involved a cohort of 716 CPP patients, among whom 71 individuals (45 females and 26 males) were identified with 18 pathogenic mutations in the *MKRN3* gene. These individuals came from 36 different families. Eight mutations were classified as severe, including six missense mutations, one stop codon mutation, and one promoter deletion. These mutations affected 53 out of 71 patients (75%) and 26 out of 36 families (72%). Compared with other mutation types, 53 children with severe *MKRN3* mutations showed more significant bone age advance and higher serum basal LH concentrations. However, there were no differences in age of onset or menarche, height, and weight. Compared with other ICPP patients, the children with MKRN3 mutations had a shorter time from symptom onset to diagnosis and a smaller height standard deviation (SD) but higher serum basal FSH concentrations.

There have been studies abroad on the efficacy of GnRHa on CPP children caused by *MKRN3* mutations [22]. Compared to the ICPP group, the *MKRN3* mutation group had smaller bone age at diagnosis, younger age, and smaller height SD. There were no differences in hormone concentrations. After at least 2 years of GnRHa treatment, there were no differences between the *MKRN3* mutation group and the ICPP group in final height, hormone concentrations, or BMI. Obesity rates increased in both groups at the end of treatment while decreased when the subjects reached FH.

At present, there is no long-term follow-up study on the outcomes of CPP children with *MKRN3* mutations after treatment with GnRHa in Asia. In the present study, we collected data related to the initial clinical symptoms and laboratory results of 4 children with FCPP due to *MKRN3* mutations, as well as the data during and after at least 2 years of GnRHa treatment and compared them to evaluate the efficacy and safety of the GnRHa therapy.

## 2. Patients and Methods

### 2.1. Study Design and Patients

We recruited 4 CPP children (3 girls and 1 boy) with *MKRN3* mutations who were admitted to the Pediatric Endocrinology Department of Shanghai Ruijin Hospital (Shanghai, China) from June 2014 to December 2021. These children sought medical advice due to the development of secondary sexual characteristics in advance and were diagnosed as CPP meeting international standard after medical examinations [23]. All patients got normal results on magnetic resonance imaging (MRI) of the CNS and underwent the GnRH stimulation test. After questioning by the attending doctor, it was known that all of these four children had family members with early pubertal development, thus the existence of FCPP caused by genetic mutations was highly suspected. The whole-exome sequencing was performed on patients and their family members to conform *MKRN3* mutations and further Sanger sequencing exhibited the same mutation in their family members (Table 1, Appendix 1). Classification of variants (pathogenic, likely pathogenic, VUS, likely benign, and benign) has been done according to the variant interpretation guidelines of the American College of Medical Genetics and Genomics (ACMG) [24]. All participants were treated exclusively with GnRHa (3.75 mg of the long-acting depot triptorelin or leuprorelin) monthly for at least 2 years.

### 2.2. Clinical and Laboratory Data Collection

Patients were followed up every 6 months during the treatment to collect clinical data, such as height, weight, and pubertal stage, as well as laboratory data, including LH, FSH, estradiol (E2), and testosterone (T). Imaging included X-ray of the hand and wrist and ultrasound of the uterus and ovary. We use the abovementioned data to evaluate whether secondary sexual characteristics, GV, BAA, and sexual steroids concentrations are effectively controlled.

Ht-SDS was calculated using the data from the National Survey on Physical Growth and Development of Children in the Nine Cities of China performed in 2005. Those who had a BMI exceeding 1 SD were therefore classified as overweight. If BMI exceeded 2 SDs, they were classified as obese. PAH was predicted by height and bone age. Girls' TH was mid-parental height minus 6.5 cm while boys' TH was mid-parental height plus 6.5 cm. The TH range was determined as TH ± 8.5 cm [22]. Pubertal stage was evaluated according to Marshall and Tanner's method, and bone age was assessed according to the method presented by Greulich and Pyle.

### 2.3. Statistical Analysis

Statistical analysis was performed using SPSS 23.0 software (IBM, Armonk, NY, USA). Data were presented as mean ± SDs. Comparisons among mean values of numerical variables were performed using the *t*-test.

## 3. Results

### 3.1. Clinical and Hormonal Data at the First Visit

The main problems in girls were premature breast development, whereas increased testicular size and penile enlargement were found in boys. The age of onset in girls was earlier than that in boys, and they all had an advanced bone age. All patients' BMI exceeded mean values at the first visit. Among them, one girl was overweight and one girl was obese. LH peak concentrations of the GnRH stimulation test were at the pubertal range in all patients. Ultrasound of the uterus and ovary in three girls showed evident development of genital organs. More detailed clinical and hormonal data are presented in Table 2.

### 3.2. Response to at Least Two-Year GnRHa Treatment

After at least 2 years of GnRHa treatment, their average height increased by 4.5 cm/y. Basal concentrations of LH in serum decreased from 1.76 ± 0.84 to 0.64 ± 0.39 (*p*=0.095) and FSH in serum decreased from 4.89 ± 1.89 to 1.72 ± 0.42 (*p*=0.038), indicating that the secretion of gonadotropin in the anterior pituitary is inhibited effectively.

The volume of the uterus declined from 5.72 ± 2.58 to 2.12 ± 1.62 mL (*p*=0.030), and the volume of the ovaries decreased from 3.61 ± 1.67 to 0.62 ± 0.22 mL (*p*=0.007), indicating remarkable suppression of gonadal hormone production. No significant progression of the breast and testicular was found. BAA decreased from 2.45 ± 0.41 to 1.48 ± 1.02 (*p*=0.184), which indicated that the advanced bone age was not inhibited distinctly and maintained its speed of growth. Thus, we could infer that these patients' height may not reach the target value. Ht-SDS decreased slightly from 1.15 ± 0.77 to 0.70 ± 0.66 (*p*=0.186). The average GV was 0.70 ± 0.66 cm/y during treatment. Due to the lack of growth rate before treatment, it is uncertain whether GnRHa can remarkably reduce growth rate. PAH slightly increased after treatment (*p*=0.285), while no significant difference was found. Thus, it was not confirmed that GnRHa can improve their PAH. Both average BMI-SDS (*p*=0.302) and obesity prevalence (*X*^2^ = 7.188, *p*=0.029) decreased during the treatment. Patient #3 who was obese at the beginning experienced a significant reduction in BMI-SDS and was overweight after treatment (Tables 3 and 4).

### 3.3. Data During the Treatment and Follow-Up

**Figure 2:**
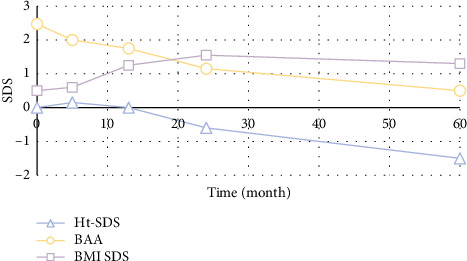
Body data of Patient 1.

**Figure 3:**
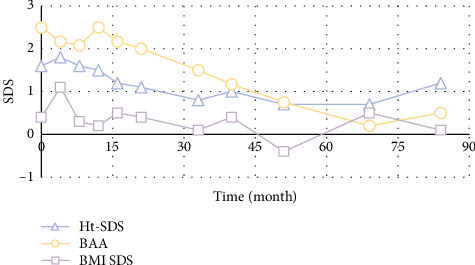
Body data of Patient 2.

The average height increase of the four subjects was 13.65 cm until the end of treatment. Patient#1 and Patient#2 have reached FH, among which the girl reached TH while the boy did not. Another two have completed the treatment recently and are still followed up every 6 months after therapy. Data collected during the therapy and follow-up are illustrated in Figures 2 and 3. It was revealed that BMI-SDS of Patient #1 increased continuously during the treatment, while it was stabilized after withdrawal. Meanwhile, his bone age advance has not been effectively inhibited, and his GV was slow. Therefore, his final height was not satisfactory and did not reach the TH range. Patient #2 received growth hormone (0.14 U/kg/d) due to the poor GV since 40th GnRHa during the therapy. After that, her GV was still inconsiderable, while her bone age was controlled well since the third year. Although her BMI fluctuated during the treatment and after withdrawal, it was generally in the normal range. Fortunately, she reached TH at the recent follow-up.

## 4. Discussion

Long-acting GnRHa has been the first-line treatment for CPP children. In the present study, we described clinical and hormonal data of 4 patients with CPP due to *MKRN3* mutations before and after treatment, as well as long-term follow-up of who completed the therapy.

During regular GnRHa treatment, all patients reached clinical and hormonal controls such as stagnation or regression of secondary sexual characteristics, decrease of serum gonadal hormone concentrations, and reduction of gonad volume. These findings presented regression of secretion of pituitary and gonadal development, indicating that long-term GnRHa treatment was satisfactory.

Whether the height can be effectively improved after treatment is the most important problem that particularly requires scholars' attention. Studies showed that those patients with *MKRN3* mutations were younger at the first visit than ICPP ones [5, 22]. However, there was no significant difference in mean values of FH and TH, indicating that *MKRN3* defects might affect the age of onset but not FH [22, 25]. Children with very early onset CPP (< 6 years old) mainly come to a satisfactory FH, which may reach the TH range or significantly exceed PAH at the beginning of therapy [10, 26–28]. In the present study, all patients, except for Patient #2, entered puberty at the age of over 6 years. Despite the use of growth hormone during the GnRHa therapy, Patient #2 is also the only one who has reached TH at present, which is consistent with the previous study showing that the combination of GnRHa with GH produced better height gains than GnRHa alone in patients with CPP [29]. In particular, Patient #1 had late onset age and treatment, significantly advanced bone age, as well as progression of bone age and BMI-SDS, poor GV during treatment; thus, the FH was not ideal, and he did not reach the TH range. GV during therapy and height at withdrawal are positively associated with FH, while they cannot be used as independent factors for indicating the time of termination of the treatment [30, 31]. For a child with unexplained deceleration of growth rate, attention might be paid to stop treatment or to introduce adjunct therapies. Although their mean PAH slightly increased after treatment, no statistically significant difference was found. It should be clarified whether the poor improvement of PAH is related to the age at onset. A longer follow-up study is required to indicate whether GnRHa can improve FH of CPP children with *MKRN3* mutations.

A growing body of evidence demonstrated that obesity advances the age of the onset of puberty and reduces the response of LH to GnRH [1, 32–34]. There has been concern that GnRHa could increase the BMI. A previous research showed that girls with ICPP were frequently obese at the onset of GnRHa therapy, which could be due to the hormonal changes, and their obesity was neither long-lasting nor related to GnRHa therapy. Ramos et al. also found that the prevalence of obesity increased at the end of treatment, while it decreased at the FH [22]. On the contrary, 2 years of GnRHa therapy may decrease the BMI. The downward trend was accompanied by a complete suppression of gonadotropin secretion, which persisted in the follow-up after withdrawal [35]. In our study, all patients' BMI were all above the average at the onset, which is frequently observed in CPP patients. Additionally, it was revealed that both average BMI-SDS and obesity prevalence decreased after 1 year of treatment. However, the changes in BMI-SDS during the treatment and follow-up in Patient #1 were consistent with Ramos et al.'s findings. A large-scale study is essential to clearly indicate whether long-term GnRHa treatment may increase the BMI.

## 5. Conclusion

In summary, GnRHa is effective for CPP patients with *MKRN3* mutations including inhibiting gonadal development and gonadal hormone concentrations. Comprehensive assessment of efficacy and safety of GnRHa therapy still requires a large-scale, long-term follow-up study.

## Figures and Tables

**Table 1:** Genetic data of four patients with MKRN3 mutations.

Patient	Position	cDNA	Protein	ACMG criteria
1	Noncoding area	−81C > T	—	PS3 + PM2 + PP1
2	Coding area	c.382_401del	p.T128A fs∗10	PVS1 ++ PM2 + PS3
3	Coding area	c.277G > A	p.G93S	PS3 + PM2 + PP1
4	Coding area	c.980G > T	p.R327L	PM2 + PP1 + PS3

*Note:* ACMG, American College of Medical Genetics and Genomics.

**Table 2:** Clinical and hormonal data of four patients with CPP due to MKRN3 mutations.

	Patient		1	2	3	4
	Sex		M	F	F	F
At onset	CA	Years	8.83	5.67	7.00	6.83

At the first visit	CA	Years	9.83	6.00	9.08	7.58
	BA	Years	12.30	8.50	11.00	10.50
	BAA	Years	2.47	2.50	1.92	2.92
	Ht-SDS		0	1.60	1.60	1.40
	BMI SDS		0.50	0.40	2.60	1.70
	Tanner stage		G2-3	B2	B3	B3
	Pubic hair stage		3-4	1	1	1

Hormonal profile	Basal LH	IU/L	2.18	0.31	2.50	1.81
	Peak LH	IU/L	38.86	18.34	13.02	23.27
	Basal FSH	IU/L	6.59	4.02	6.33	2.64
	Peak FSH	IU/L	15.92	17.22	13.14	11.45
	E2	pg/mL	< 10	23	32	< 10
	T	ng/mL	5.50	< 0.08	0.14	< 0.08
	IGF-1	ng/mL	596	377	430	286

Genital organs	Volume of the uterus	mL	—	4.09	8.70	4.39
	Volume of the ovaries	Left (mL)	—	1.95	4.64	3.86
		Right (mL)	—	1.21	4.58	5.43
	Length of the penis	cm	5	—	—	—

*Note:* Ht-SDS, height-based standard deviation scores corresponding to the chronological age; IGF-1, insulin-like growth Factor 1.

Abbreviations: BA, bone age; BAA, bone age advance; BMI-SDS, body mass index–standard deviation scores; CA, chronological age.

**Table 3:** The effects of GnRHa treatment on clinical features.

Patient/sex	At the first visit		After 2 years	
	BAA	Ht-SDS	BAA	Ht-SDS
1/M	2.47	0	2.84	0
2/F	2.5	1.6	0.34	0.6
3/F	1.92	1.6	1.42	1.6
4/F	2.92	1.4	1.34	0.6

*Note:* Ht-SDS, height-based standard deviation scores corresponding to the chronological age.

Abbreviation: BAA, bone age advance.

**Table 4:** Comparison of clinical and hormonal data before and after GnRHa treatment.

	At the first visit (mean ± SD)	After GnRHa treatment (mean ± SD)	*p*
Ht-SDS	1.15 ± 0.77	0.70 ± 0.66	0.186
GV (cm/y)	NA	6.28 ± 1.98	—
PAH	156.12 ± 1.03	159.75 ± 2.63	0.285
BAA	2.45 ± 0.41	1.48 ± 1.02	0.184
BMI (kg/m^2^)	18.80 ± 2.96	19.4 ± 2.37	0.580
BMI-SDS	1.30 ± 1.04	0.80 ± 1.09	0.302
Obesity (%)	25	0	0.029
Volume of the uterus (mL)	5.72 ± 2.58	2.12 ± 1.62	0.030
Volume of the ovaries (mL)	3.61 ± 1.67	0.62 ± 0.22	0.007
Basal LH (IU/L)	1.76 ± 0.84	0.64 ± 0.40	0.095
Basal FSH (IU/L)	4.89 ± 1.89	1.72 ± 0.42	0.038

*Note:* Ht-SDS, height-based standard deviation scores corresponding to the chronological age.

Abbreviations: BAA, bone age advance; BMI, body mass index; BMI-SDS, body mass index–standard deviation scores; GV, growth velocity; NA, not available; PAH, predicted adult height.

## Data Availability

Due to personal privacy concerns, all data (including laboratory, genetic, and body measurement data) have not been uploaded in the Internet. The data related to the experiment have been described in the text. If there are any other needs, please contact us by email.

## Supporting Information

Pedigrees of four subjects and their mutation sites are shown. Specific information is provided in the figure and the annotation below. LH reference ranges are presented at different Tanner stages.

## References

[1] Livadas S., Chrousos G. P. (2019). Molecular and Environmental Mechanisms Regulating Puberty Initiation: An Integrated Approach. *Frontiers in Endocrinology*.

[2] Lomniczi A., Castellano J. M., Wright H., Selcuk B., Ojeda S. R. (2014). Gene Networks, Epigenetics and the Control of Female Puberty. *Springer International Publishing*.

[3] Parent A. S., Teilmann G., Juul A., Skakkebaek N. E., Toppari J., Bourguignon J. P. (2003). The Timing of Normal Puberty and the Age Limits of Sexual Precocity: Variations Around the World, Secular Trends, and Changes After Migration. *Endocrine Reviews*.

[4] De Vries L., Kauschansky A., Shohat M., Phillip M. (2004). Familial Central Precocious Puberty Suggests Autosomal Dominant Inheritance. *Journal of Clinical Endocrinology and Metabolism*.

[5] Seraphim C. E., Canton A. P. M., Montenegro L. (2021). Genotype-Phenotype Correlations in Central Precocious Puberty Caused by MKRN3 Mutations. *Journal of Clinical Endocrinology and Metabolism*.

[6] Abreu A. P., Dauber A., Macedo D. B. (2013). Central Precocious Puberty Caused by Mutations in the Imprinted Gene MKRN3. *New England Journal of Medicine*.

[7] Abreu A. P., Macedo D. B., Brito V. N., Kaiser U. B., Latronico A. C. (2015). A New Pathway in the Control of the Initiation of Puberty: The MKRN3 Gene. *Journal of Molecular Endocrinology*.

[8] Comite F., Cassorla F., Barnes K. M. (1986). Luteinizing Hormone Releasing Hormone Analogue Therapy for Central Precocious Puberty. Long-Term Effect on Somatic Growth, Bone Maturation, and Predicted Height. *JAMA*.

[9] Carel J. C., Eugster E. A., Rogol A. (2009). Consensus Statement on the Use of Gonadotropin-Releasing Hormone Analogs in Children. *Pediatrics*.

[10] Fuqua J. S. (2013). Treatment and Outcomes of Precocious Puberty: An Update. *Journal of Clinical Endocrinology and Metabolism*.

[11] Jiang L. Q., Zhou Y. Q., Yuan K., Zhu J. F., Fang Y. L., Wang C. L. (2021). Rare Mutation in MKRN3 in Two Twin Sisters With Central Precocious Puberty: Two Case Reports. *World Journal of Clinical Cases*.

[12] Yin X., Wang J., Han T. (2021). A Novel Loss-of-Function MKRN3 Variant in a Chinese Patient With Familial Precocious Puberty: A Case Report and Functional Study. *Frontiers in Genetics*.

[13] Varimo T., Iivonen A. P., Känsäkoski J. (2021). Familial Central Precocious Puberty: Two Novel MKRN3 Mutations. *Pediatric Research*.

[14] Zzubkova N. A., Kolodkina A. A., Makretskaya N. A. (2021). Clinical and Molecular Genetic Features of 3 Family Cases of the Central Precocious Puberty, Due to MKRN3 Gene Defects. *Problemy Endokrinologii*.

[15] Gernay C., Brachet C., Boros E., Tenoutasse S., Libioulle C., Heinrichs C. (2022). Six Novel Variants in the MKRN3 Gene Causing Central Precocious Puberty. *Journal of the Endocrine Society*.

[16] Magnotto J. C., Mancini A., Bird K. (2023). Novel MKRN3 Missense Mutations Associated With Central Precocious Puberty Reveal Distinct Effects on Ubiquitination. *Journal of Clinical Endocrinology and Metabolism*.

[17] Liu J., Li T., Peng M. (2023). The Key Roles of Makorin RING Finger Protein 3 (MKRN3) During the Development of Pubertal Initiation and Central Precocious Puberty (CPP). *Current Molecular Medicine*.

[18] Kırkgöz T., Kaygusuz S. B., Alavanda C. (2023). Molecular Analysis of MKRN3 Gene in Turkish Girls With Sporadic and Familial Idiopathic Central Precocious Puberty. *Journal of Pediatric Endocrinology & Metabolism*.

[19] Chen Z., You Q., Wang J. (2024). The Functional Study of a Novel MKRN3 Missense Mutation Associated With Familial Central Precocious Puberty. *American Journal of Medical Genetics, Part A*.

[20] Karaman V., Karakilic-Ozturan E., Poyrazoglu S. (2024). Novel Variants Ensued Genomic Imprinting in Familial Central Precocious Puberty. *Journal of Endocrinological Investigation*.

[21] Lu W., Wang J., Li C., Sun M., Hu R., Wang W. (2018). A Novel Mutation in 5′-UTR of Makorin Ring Finger 3 Gene Associated With the Familial Precocious Puberty. *Acta Biochimica et Biophysica Sinica*.

[22] Ramos C. O., Macedo D. B., Canton A. P. M. (2020). Outcomes of Patients With Central Precocious Puberty Due to Loss-of-Function Mutations in the MKRN3 Gene After Treatment With Gonadotropin-Releasing Hormone Analog. *Neuroendocrinology*.

[23] Cheuiche A. V., da Silveira L. G., de Paula L. C. P., Lucena I. R. S., Silveiro S. P. (2021). Diagnosis and Management of Precocious Sexual Maturation: An Updated Review. *European Journal of Pediatrics*.

[24] Richards S., Aziz N., Bale S. (2015). Standards and Guidelines for the Interpretation of Sequence Variants: a Joint Consensus Recommendation of the American College of Medical Genetics and Genomics and the Association for Molecular Pathology. *Genetics in Medicine*.

[25] Pagani S., Calcaterra V., Acquafredda G. (2020). MKRN3 and KISS1R Mutations in Precocious and Early Puberty. *Italian Journal of Pediatrics*.

[26] Kaplowitz P. B., Backeljauw P. F., Allen D. B. (2018). Toward More Targeted and Cost-Effective Gonadotropin-Releasing Hormone Analog Treatment in Girls with Central Precocious Puberty. *Hormone Research in Paediatrícs*.

[27] Paul D., Conte F. A., Grumbach M. M., Kaplan S. L. (1995). Long-Term Effect of Gonadotropin-Releasing Hormone Agonist Therapy on Final and Near-Final Height in 26 Children With True Precocious Puberty Treated at a Median Age of Less Than 5 Years. *Journal of Clinical Endocrinology and Metabolism*.

[28] Cassio A., Cacciari E., Balsamo A., Bal M., Tassinari D. (1999). Randomised Trial of LHRH Analogue Treatment on Final Height in Girls With Onset of Puberty Aged 7.5–8.5 Years. *Archives of Disease in Childhood*.

[29] Shi Y., Ma Z., Yang X., Ying Y., Luo X., Hou L. (2022). Gonadotropin-Releasing Hormone Analogue and Recombinant Human Growth Hormone Treatment for Idiopathic Central Precocious Puberty in Girls. *Frontiers in Endocrinology*.

[30] Lazar L., Padoa A., Phillip M. (2007). Growth Pattern and Final Height After Cessation of Gonadotropin-Suppressive Therapy in Girls With Central Sexual Precocity. *Journal of Clinical Endocrinology and Metabolism*.

[31] Pasquino A. M., Pucarelli I., Accardo F., Demiraj V., Segni M., Di Nardo R. (2008). Long-Term Observation of 87 Girls With Idiopathic Central Precocious Puberty Treated With Gonadotropin-Releasing Hormone Analogs: Impact on Adult Height, Body Mass Index, Bone Mineral Content, and Reproductive Function. *Journal of Clinical Endocrinology and Metabolism*.

[32] Herman-Giddens M. E., Kaplowitz P. B., Wasserman R. (2004). Navigating the Recent Articles on Girls’ Puberty in Pediatrics: What Do We Know and Where Do We Go From Here?. *Pediatrics*.

[33] Rosenfield R. L., Lipton R. B., Drum M. L. (2009). Thelarche, Pubarche, and Menarche Attainment in Children With Normal and Elevated Body Mass Index. *Pediatrics*.

[34] Fu J. F., Liang J. F., Zhou X. L. (2015). Impact of BMI on Gonadorelin-Stimulated LH Peak in Premenarcheal Girls With Idiopathic Central Precocious Puberty. *Obesity*.

[35] Arrigo T., De Luca F., Antoniazzi F. (2004). Reduction of Baseline Body Mass Index Under Gonadotropin-Suppressive Therapy in Girls With Idiopathic Precocious Puberty. *European Journal of Endocrinology*.

