# Supplementary material for: Outcomes of Patients With Familial Central Precocious Puberty due to Mutations of MKRN3 Gene After Treatment With Gonadotropin-Releasing Hormone Agonist
Source: Int J Endocrinol. 2025 Nov 21;2025:5609749. doi: 10.1155/ije/5609749 (PMC12662689; doi:10.1155/ije/5609749)
Supplement: Supporting Information — Additional supporting information can be found online in the Supporting Information section. [file 5609749.f1.pdf]

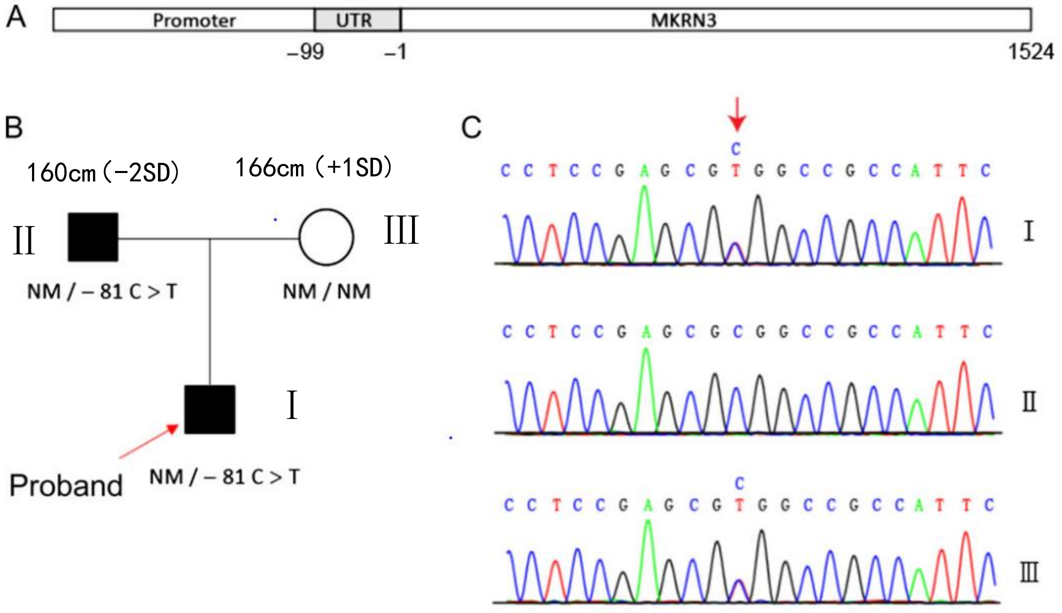

Figure 1: A mutation in the 5 'UTR region of the MKRN3 gene in patient 1.

(A) Schematic diagram of the human MKRN3 gene, including the promoter, 5' -UTR, and coding regions. (B) A family with a newly discovered -81C>T mutation in the 5 '-UTR region of the MKRN3 gene. The arrow points to the first affected individual, where squares represent males, circles represent females, black indicates affected individuals, white indicates healthy individuals, and NM indicates no mutation. (C) Partial sequencing chromatogram of the 5' -UTR region of the MKRN3 gene, with the arrow pointing to the-81C>T mutation.

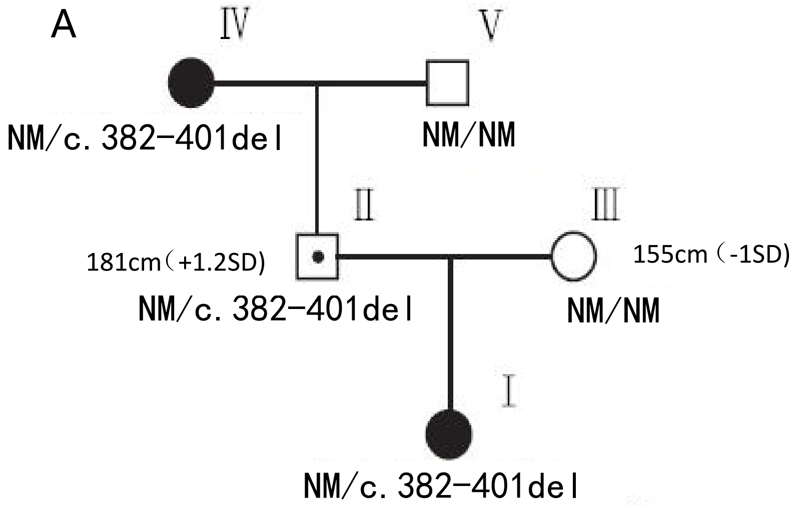

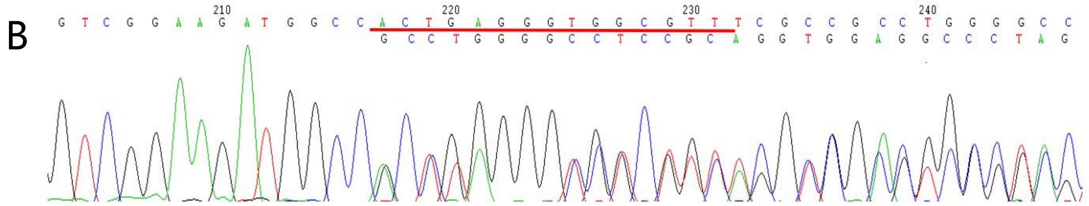

Figure 2:A mutation in the coding region of MKRN3 in patient 2.

(A) A family with a newly discovered frameshift + deletion mutation in the coding region of MKRN3 gene. (B) The chromatogram of partial sequencing of the MKRN3 gene in the first witness, with the wavy line indicating a frameshift + deletion mutation.

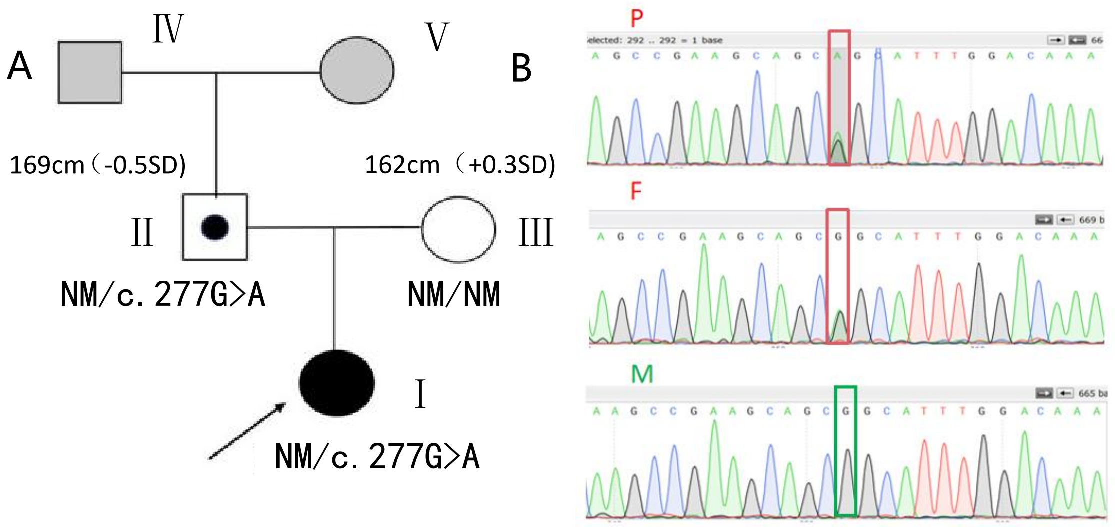

Figure 3: A mutation in the coding region of MKRN3 in patient 3.

(A)A family with c.277G>A/p.G93S mutation found in the coding region of MKRN3 gene. (B) Partial sequencing chromatogram of MKRN3 gene, with c.277G>A mutation highlighted .

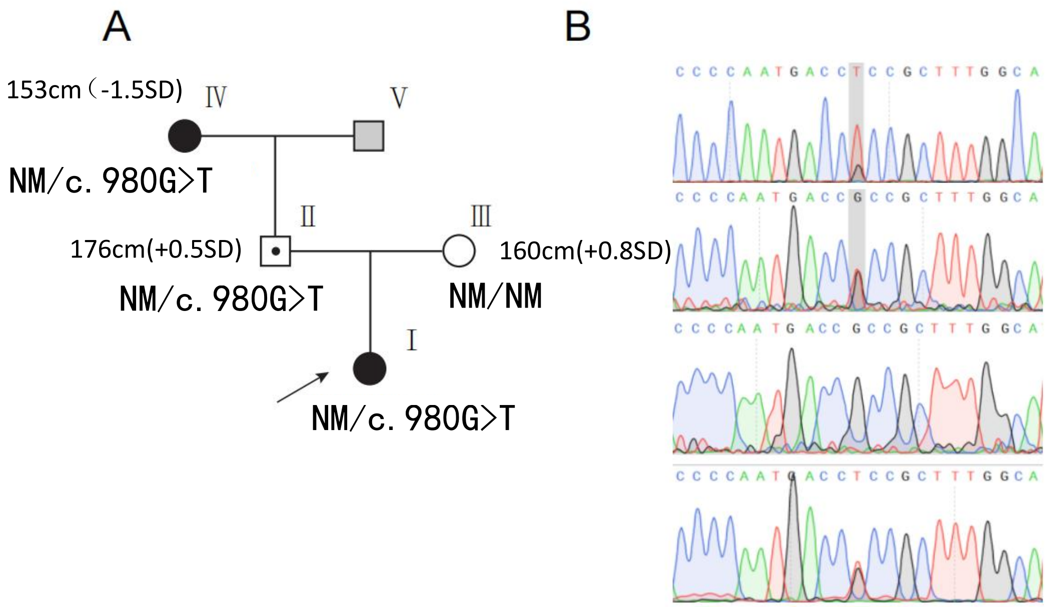

Figure 4:A mutation in the coding region of MKRN3 in patient 4.

(A) A family with c.980G>T/p.R327L mutation found in the coding region of MKRN3 gene. (B) Partial sequencing chromatogram of the coding region of MKRN3 gene.

LH levels are shown in the below table.

Tanner	Male	Female
1	0.02-0.03U/L	0.02-0.18U/L
2	0.2-4.9U/L	0.02-4.7U/L
3	0.2-5.0U/L	0.1-12.0U/L
4	0.4-7.0U/L	0.4-11.7U/L
5	1.5-9.0U/L	Follicular phase 18-49U/L
		Luteal phase 2-11U/L
